# The Moderating Role of Autonomous Motivation on the Relationship between Subjective Well-Being and Physical Health

**DOI:** 10.1371/journal.pone.0126399

**Published:** 2015-05-05

**Authors:** Ivana Marcinko

**Affiliations:** Department of Psychology, Faculty of Humanities and Social Sciences, University of J. J. Strossmayer Osijek, Osijek, Croatia; Harbin Medical University, CHINA

## Abstract

The purpose of this study was to investigate the moderator effects of autonomous motivation on the relationship between subjective well-being and physical health. Using a cluster sampling approach 486 students (403 female and 83 male students) were included in this study. Subjective well-being, physical health and autonomous motivation were determined by self-report measures. Data were analysed using hierarchical regression analysis and analysis of variance. The results show that high self-determination moderates the relationship between high subjective well-being and physical health. Accordingly, the best physical health was reported by participants who had high level of subjective well-being and whose behaviours were self-determined. Additional analyses have shown that the moderating effect of self-determination is based on the moderational impact of autonomous motives and not the controlling ones. Additionally, whether autonomous motivation moderates the relationship between components of subjective well-being and physical health was also tested. The findings have shown that autonomous motives moderate relationship between physical health and one component of well-being, positive affect. Consequently, a good physical health was found among participants with high positive affect and behaviours regulated by high degree of autonomous motives. Conclusion which can be drawn from these findings is that if an individual behaves autonomously then it can contribute to positive mind—body influences and support their own health.

## Introduction

If we look at the vast amount of research conducted over the past decades in the area of clinical and health psychology, it could be noted that mainstream scientist have been focused on the relationship between negative psychological states, mental and somatic health. Only recently, focus has been shifted to the impact of positive psychological states on health. In this context large number of articles has been written on how subjective well—being or happiness, as is more commonly referred to, relates to physical health. Some of the studies were correlational in nature and others longitudinal. Physical health was measured both, objectively using morbidity and mortality rates or medical examination, and subjectively, using self-reports. In general, research has shown invaluable evidence that subjective well—being is associated to physical health.

A review of the correlational findings on the relationship between health and subjective well-being show that correlational coefficients vary between 0.10 and 0.45 and tend to be independent of sociodemographic variables and personality [1, 2]. Correlations tend to be larger in studies with ill people rather than healthy ones. Also, higher coefficients tend to be present between subjective well—being and self-assessed health in comparison to objective indicators of health.

As for the relationship between subjective well—being and objective health there are an extensive number of studies that point to conclusion that well—being contributes to longevity. These studies are longitudinal in nature where a certain group of people whose well-being had been measured at the beginning of the study, were followed for some time. The majority of these studies lasted between seven and ten years although there are some that covered the entire adulthood. Research conducted on people who living with serious medical conditions (e.g. breast cancer, melanoma or heart condition) have shown that happiness doesn’t contribute to longevity of that population. However, happiness seems to extend a life span of healthy people [1]. One of the most famous studies with healthy subjects has been conducted on a sample of nuns entering a convent in their early twenties. Their level of well-being was taken from their autobiographical notes which they made at the beginning of the life there. What was found sixty years later was that positive emotional content of their autobiographical notes strongly relates to survival rate during their old age [3]. Levy, Slade, Kunkel and Kasl [4] have also investigated the relationship between subjective well—being and longevity of people who were 50 years old and over and found a positive association between those variables. In their study happy people on average lived 7.5 years longer than people who reported to be less happy. According to some research these relationships can be explained by immunological system functioning precisely, lymphocyte functioning. Bartrop, Lazarus, Luckhurst, Kiloh and Penny [5] have found depressed lymphocyte function among bereaved groups. When bereavement was related to the loss of a life partner functioning of T and B cells within the immune system were disturbed for months after and had to be artificially controlled so their usual functions were normally performed [6, 7]. Aside from this, there are indications that mental states influence cellular changes which in turn, affect our physical health. Anti-ageing research reveals that chronic stress impacts physical health by modulating the rate of cellular ageing. It was shown that the greater the psychological stress a person is living, the greater oxidative stress occurs and disturbance in telomerase activity contributing to many illnesses which are accelerated by ageing [8].

A link between subjective well—being and subjective measures of physical health is also equally well documented among older and younger generations. In a study by Cho, Martin, Margrett, MacDonald and Poon [9] positive association between psychological well—being and subjective health was found among the oldest—old adults. Mechanic and Hansell [10] conducted a longitudinal study with adolescents investigating the relationship between adolescent competence, psychological well—being and self—assessed physical health. Results have shown that a greater sense of physical health was determined by higher well—being measured by low level of depressive symptoms in adolescents. One other longitudinal study with a sample of participants aged 18 years old and older registered that happier people tend to report better physical health even when the effects of baseline health, sociodemographic variables and several health behaviors have been controlled [11].

Although health research indicates that subjective well-being is important for physical health it is well recognized by now that not all types of well-being contributes to it equally. The focus was on how eudaimonia, or well-being which arises from autonomous activities and which reflects the development of one's own potential as opposed to hedonia, or a form that reflects the sum of individual positive experiences impact somatic health. Ryff, Singer and Love [12] conducted a research whereby these relationships were tested. They used Ryff’s [13] six-factor model and measurement scales (autonomy, environmental mastery, personal growth, positive relations with others, purpose in life and self-acceptance) while criterion variables were neuroendocrinological, immune, cardiovascular factors, and the amount of rapid eye movement (REM) sleep. The results have shown that eudaimonia is related to a series of factors that contribute to positive health outcomes, while hedonia is only associated with one. Participants with higher levels of meaning in life and personal growth had lower levels of daily cortisol, a hormone that positively correlates with stress. Purpose in life was associated with a lower number of pro-inflammatory cytokines, particularly interleukin-6. Positive relations with others, personal growth and purpose in life correlated negatively with glycosylated hemoglobin, weight, waist and hip ratio, while a positive correlation was confirmed with "good" high density lipoprotein (HDL) cholesterol. Environmental mastery was associated with a greater amount of sleep and a faster onset of REM sleep. In contrast, hedonia was related only to "good" HDL cholesterol. Other studies also suggest that eudaimonia has a much greater positive impact on health than hedonia. Fredrickson et al. [14] investigated the relationship between conserved transcriptional response to adversity (CTRA) and hedonia and eudaimonia. CTRA involves an increased expression of genes involved in the inflammatory processes and a decreased expression of genes involved in antibody synthesis and antiviral processes under chronic exposure to adverse events, which can ultimately lead to cardiovascular, neurodegenerative conditions and infections [15, 16]. Their study demonstrated that people with high hedonic well-being have up-regulated CTRA while those with high eudaimonic well-being have down-regulation of CTRA. Studies where health was measured by self-report questionnaires point to the same conclusion. Miguelon and Vallerand [17] found that eudaimonia contributes to self-rated physical health, while the impact of hedonia was found to be insignificant. According to some, positive effects which eudaimona has on health can be explained by its stress-buffering effects. Friedman et al. [18] investigated the moderating role of eudaimonia on the association between sleep efficiency and immune functions in a national sample. They found that subjects with sleep problems had deviant immune markers whilst that was not the case for participants with high eudaimonia. The moderating role of eudaimonia was also examined in the relationship between socioeconomic inequality and glycosylated hemoglobin (HbA1c), an indicator of glycemic control. It was found that eudaimonia moderates the relationship between income and HbA1c in a way that low eudaimonia amplified negative effects of low income on HbA1c, raising values which indicate a poorer control of blood glucose levels. However if eudaimona levels were high, no difference in HbA1c levels were found between low-socioeconomic and high-socioeconomic status groups [19].

Although scientific research in the field of health suggests variables which could explain discrepancies, self-determination theory (SDT) [20] explains these different outcomes by the motives. Theory posits that motivational aspects of behaviour contribute to individual differences in well—being as well as to the diversity of health outcomes. In order to understand this proposition, one has to be clear as to what autonomy means. Within the SDT framework, autonomy or self-determination is one of the key concepts. On one hand, autonomy represents motivational processes governed by the authentic part of the self. A greater degree of autonomy indicates that behaviour is in accordance with deeply held values, needs or goals of an individual rather than a reflection of the pressures which are experienced as threatening and controlling. On the other hand, autonomy is also described as a psychological need that has to be satisfied in order for growth, integrity and the health of a person to be ensured [21].

SDT proposes that all goal-directed activities can be differentially autonomous. The theory describes four motivating styles which differ according the extent to which the regulations are autonomous thereby integrated with a person's sense of self. Externally motivated and introjected regulation represent controlled forms of motivation by which people are under the influence of the various pressures that may be present as external (society, other people) or internal factors (tensions that arise from within oneself). For them, there is a distinctive feeling that an activity has to be done in certain way. Identified and integrated regulation represents autonomous forms of motivation which are characterized by a desire to self-initiate an experience and the ability to choose how to behave. When a behaviour is autonomously motivated it means a person is behaving in line with their own desires and an authentic part of him/herself [22].

Since people sometimes behave in accordance with their own needs, and sometimes do not, it has been proposed that variations in autonomy affect human functioning. It is argued that while autonomous behavior contributes to well-being and health, rigid and controlling behaviors are the cause of distress and behavioral pathology [23]. This premise was tested in early research mainly focusing on the association between autonomy and well-being. The majority of studies confirmed that autonomy is related to higher levels of well-being [24, 25]. It was also found that day to day variations in the satisfaction of the need for autonomy predicted daily well-being levels [26].

On the other hand, it was not addressed whether autonomy affects a relationship between subjective well-being and somatic health. It has not been examined whether the magnitude of the effect that well-being has on physical health depends on autonomy. So, the aim of this research was to examine the moderator effects of autonomy on the relationship between subjective well-being and physical health. As a fulfillment of need for autonomy is considered innate to people and it is argued that it exerts positive effects when fulfilled it is expected it will positively influence the wellbeing-health relationship. Since moderator affects the direction and/or strength of a relationship between two variables [27] it is anticipated that high autonomy will amplify positive effects of high well-being on physical health. Specifically, it is expected that participants with high well-being and highly autonomous behaviours will report better physical health in comparison to those with high well-being and behaviours regulated by a low degree of autonomy. Moderation effects are intended to be investigated under control of neuroticism, a potential confounding variable in this study, since the past research shows that this personality trait affects self-reports of well-being, subjective health measures as well as its relationship [28]. As it will be demonstrated the findings indicate that experiential autonomy moderates the relationship between psychological and physical aspects of human functioning.

## Materials and Methods

### Ethics statement

This study was approved by the Ethic Committee for Research with Human Subjects, Faculty of Humanities and Social Sciences, University of J. J. Strossmayer in Osijek. All the participants received verbal and written information about the study and signed an informed consent form.

### Participants and procedure

Students from the University of J. J. Strossmayer in Osijek participated in this study. Cluster sampling approach was used in order to get a representative sample of students. This process began by randomly selecting three Faculties (Faculties of Humanities and Social Sciences, Economics and Teacher Education) from the pool of the existing ones within the University. Next, five Departments from the selected Faculties were randomly chosen, followed by the sampling of individual classes until the desired number of participants had been reached. The research was conducted at a single point in time.

At the beginning of the session participants received verbal and written information on the purpose of the study and their rights before voluntary consent has been obtained. Then they were given a questionnaire packet which included questions about gender, age and general health along with all other instruments. The general instructions were then given regarding how to fill out the questionnaires as well as the assistance they would be given if they would need help. After that, they were asked to answer questions regarding gender, age and general state of health. The question of general state of health was included in order to eliminate results of participants that live with some kind of chronic illness or are exposed to a long-term therapy. It is believed that the answers of those participants would be biased which would obscure the true relationship between measured constructs. Personal goal assessment followed within which the experimenter read the instructions and asked the participants to think of the three goals they were trying to achieve in the last six months and then to write them down. After that the assessment of the goals' motives and well-being associated with it took place. Participants were asked to fill in the scales attributed to each goal (PANAS, single-item satisfaction scale, scale of autonomous/controlling motives) while keeping the focus on the goal. In that way measures of motives as well as positive and negative emotions and satisfaction attached to the goals were obtained. Once they did that, subjects were told they can continue to fill out the physical health questionnaire and neuroticism scale.

In total, 511 participants filled out the questionnaire packet. However, 25 students who completed the questionnaires reported to suffer from some form of chronic illness so their results were eliminated from the sample. Thus, the final sample consisted of 486 students of which 403 (82.9%) were females and 83 (17.1%) were males. The average age of participants was 22, ranging from 18 to 36 years.

### Measures

General health was examined with the question: "Do you suffer from a chronic disease (e.g. asthma, diabetes, arthritis, etc.)?" to which the participant responds by choosing between "yes/no" answers. In order to measure well-being, physical health and neuroticism the following instruments were used:

#### Personal goals

The goal assessment procedure was based on the personal projects model [29] but the instructions were adjusted to the requirements of this research. The instructions were: "Goals are something that people think about, plan, implement through action, and sometimes (though not always) bring to a conclusion and are successful in it. They may be more or less difficult to achieve, may take more or less time and can be of varying interest or importance." The experimenter then listed several goals to serve as an example and instructed participants to think about the three most important goals they have been trying to achieve during the last six months and to write them down. As participants were generating their own list of goals the greater ecological validity of the results has been achieved. This procedure has been originally adapted from the assessment of personal projects [29] by Sheldon and Kasser [30] and successfully used in many studies on the role of motives in well-being [17].

#### Scale of autonomous/controlling motives [31]

Autonomous and controlling motives were assessed by four items that measure four regulatory styles: intrinsic, identified, introjected and external. These four regulatory styles represent a continuum of autonomous-controlling motivation. Items that measure autonomous motives were: "I aspire to this because I think it is really important to have a goal like this" (identified reason) and "I aspire to this because of the fun and pleasure that this activity gives me—my main reason is my interest in this experience" (intrinsic reason). Items that examined the controlling reasons were: "I aspire to this goal because I will feel ashamed, guilty or upset if I do not do it" (introjected reason) and "I aspire to this goal because someone expects this of me or the situation imposes it on me" (external reason). Self-report ratings for each item were made on a seven point scale (from 1—not at all for this reason to 7—absolutely for this reason). The degree of self-determination or the relative autonomy index (RAI), associated with each goal is calculated using the formula: 2x intrinsic + identified - introjected - 2x extrinsic [32]. The total degree of self-determination is calculated by summing up the RAI results for all three goals. Autonomous motives related to each goal are calculated by adding up the responses for items that examine identified and intrinsic reasons while controlling motives are calculated summing the responses for items that measure introjected and extrinsic reasons. A single score of autonomous (controlling) motives is computed by summing responses from all autonomous (controlling) items obtained in relation to the three goals. This procedure has been used in prior work on goal motives [33] and allowed us the advantage of assessing the independent effect of the RAI along with independent role of autonomous and controlling motives on the relationship between subjective well-being and physical health.

#### Subjective well-being

Subjective well-being as a construct can be defined as global assessment of one's life from the subjective point of view of a person and it is comprised of cognitive and affective component [34, 35]. The cognitive component is evaluative judgment an individual makes about their life as a whole. The affective component refers to a judgment how enjoyable life is in terms of prevalence of positive or negative emotions. As such, subjective well-being is commonly measured with instruments that assess these components. In this study Positive and Negative Affect Schedule (PANAS) has been used for purposes of measuring affective aspects of well-being while the single—item life satisfaction scale was employed for the assessment of cognitive component.

#### PANAS [36]

PANAS is an instrument that measures two superordinate dimensions of emotion: positive affect and negative affect. This instrument comprises a list of 20 adjectives, of which 10 measure positive emotions (e.g. careful, excited) and 10 negative emotions (e.g. irritable, frightened). Ratings by participants were made on five-point scale ranging from 1 (very little or not at all) to 5 (extremely) bearing in mind the particular goal which a participant is trying to achieve. PANAS was translated and adapted for the Croatian language in one of earlier studies [37]. The reliability of the positive affect scale determined in our study ranges from .84 to .89, and between .90 and .92 for the negative affect scale.

#### Single-item life satisfaction scale [38]

A single-item life satisfaction scale is actually single question widely used to determine global life satisfaction [38]. For the purposes of this research the content of this question was changed into: "How satisfied are you in general with the achievement of this goal?" on which participants responded by choosing an answer from seven-point Likert scale (1-not satisfied at all to 7-I am completely satisfied).

These measures are widely used and well-validated instruments. In the early research on subjective well-being, researchers studying this construct were mainly using single item measures which although having satisfactory psychometric properties cannot be compared with the psychometric characteristics of multi-item measures. For this reason PANAS has been chosen for assessment of emotional component of subjective well-being while the multi-item life satisfaction scale [39] commonly used for assessment of cognitive component, was not acceptable. Bearing in mind that we were interested in measuring satisfaction with the goals, a single-item measure seem to be more appropriate.

These instruments were administered three times, once for each goal. Total score for the positive affect (negative affect or satisfaction) associated with the goals that one is trying to accomplish is calculated by adding up the responses from the positive affect (negative affect or satisfaction) scale obtained in relation to the three goals. A higher score indicates a higher prevalence of positive affect (negative affect or satisfaction) associated with these goals.

The composite measure of well-being is calculated by adding the scores obtained from the positive affect scale, the inverted results of the negative affect scale with the results of the single-item life satisfaction scale obtained in connection with the three goals.

#### Scales of physical health from the Short form 36 Questionnaire (SF-36) [40]

The SF-36 questionnaire is a widely used tool for assessing the health of general and specific populations. It contains eight scales however, for the purpose of this research, only four scales that measure physical health were used. These scales were: a) physical functioning, b) physical role functioning, c) bodily pain and d) general health perceptions. Result of each scale was computed by summing the scores across items. The superordinate measure of physical health was expressed as an average score on four scales with the lowest possible score being 0 and the highest 100. A higher score indicates a better state of health [41]. The coefficient of internal reliability for this scale determined in present study was .86. The advantage of this measure to other well-known health status surveys is that it provides more in depth assessment of physical health status considering all the facets that constitute the core physical health measure.

#### Neuroticism scale from the Big-Five Inventory (BFI) [42]

This scale includes eight questions (e.g. I see myself as a person who is depressed, sad) on which subjects responded by choosing one of the answers from 1-strongly disagree to 5-I absolutely agree, indicating the extent to which a participant perceives themselves to be a person with this particular feature. A total score is obtained by summing the responses across the items. The BFI has been translated into Croatian language and used in previous research [43], in which the original structure of the questionnaire was confirmed. The reliability of the neuroticism scale determined in this research was .80.

## Results

Table 1 shows the descriptive statistics and Pearson correlation coefficients of the research variables. What can be seen from results of descriptive analysis is that participants in this study had higher prevalence of autonomous than controlling motives associated with goals. Bearing in mind that the results on the scales of the PANAS inventory as well as on the single-item satisfaction scale were above the point of neutrality, it is concluded that subjects of this study are happy. On the scale of satisfaction, the point of neutrality represents a state of equal satisfaction and dissatisfaction though when emotions or moods are concerned, the point of neutrality represents a state of equal representation of positive and negative emotions. Hedonism implies a higher incidence of positive compared to negative affect. Finally, the average score obtained on the physical health scale suggests that the participants in this study were healthy.

**Table 1:** Mean, standard deviation and correlations among the study variables.

	M	SD	1	2	3	4	5	6	7	8	9
1. Subjective well-being	236.31	32.39									
2. Positive affect	108.73	18.09	.76^b^								
3. Negative affect	68.26	20.84	-.80^b^	-.22^b^							
4. Satisfaction with goals	15.83	3.32	.60	.52^b^	-.32						
5. RAI	10.51	13.04	.46^b^	.32^b^	-.39^b^	.32^b^					
6. Autonomous motives	34.33	5.60	.31^b^	.44^b^	-.06	.30^b^	.54^b^				
7. Controlling motives	20.58	8.52	-.34^b^	-.08	.43^b^	-.16^b^	-.66^b^	.07			
8. Neuroticism	23.12	5.54	-.39^b^	-.16^b^	.43^b^	-.22^b^	-.10^a^	-.03	.13^b^		
9. Physical health	73.23	17.99	.30^b^	.13^b^	-.33^b^	.15^b^	.19^b^	.09^a^	-.15^b^	-.32^b^	

^a^p< .050

^b^ p< .010

The correlation matrix indicated that subjective well-being, its components and motives were significantly related to physical health. The correlations between subjective well-being and its components on one hand, and physical health on the other, were low. It ranged from .13 to -.33. Correlation coefficients between motives and physical health were also found to be low, whereby the highest correlation with health had RAI (.19).

In order to examine whether autonomous motivation moderate the association between subjective well-being and physical health, a hierarchical regression analysis was performed. Given that here it was the effect of the interaction between motives and well-being on physical health that was being tested, predictor and moderator variables were centered in order to avoid multicollinearity [44]. Neuroticism, motives and subjective well-being were predictors, while physical health was the criterion variable. As moderational effects needed to be tested after controlling for the effects of neuroticism, this personality trait was entered in the first step of the analysis while RAI was entered in the second, subjective well-being in the third, and the interaction between RAI and subjective well-being was entered in the fourth step. In additional analyses autonomous and controlling motives were included instead of RAI. The results of these analyses can be found in Tables 2 and 3. Interaction between RAI and well-being has found to be significant, which confirms that self-determination moderates the relationship between subjective well-being and physical health. Interaction between autonomous motives and subjective well-being was also significant which confirms the autonomous motives in the role of the moderator of the relationship between subjective well-being and somatic health.

**Table 2:** The results of regression analyses in which RAI was tested as the moderator of the relationship between subjective well-being and physical health.

PHYSICAL HEALTH			
Predictors	Beta ^c^	R^2^ ^d^	ΔR2 ^e^
**1. step**			
Neuroticism	-.32^b^	.10^b^	
**2. step**			
RAI	.17^b^	.13	.03^b^
**3. step**			
Subjective well-being	.18^b^	.15	.02^b^
**4. step**			
RAI x Subjective well-being	.10^a^	.16	.01^a^

^a^ p< .010

^b^ p<.001

^c^ Beta-how many standard deviations a dependent variable will change, per standard deviation increase in the predictor variable

^d^ R^**2**^- proportion of variance in the criterion variable explained by the predictor variable

^e^ ΔR2- difference in R^2^ between previous and current step of the analysis

**Table 3:** The results of regression analyses in which autonomous and controlling motives were tested as the moderators of the relationship between subjective well-being and physical health.

PHYSICAL HEALTH			
Predictors	Beta^d^	R^2^ ^e^	ΔR2^f^
**1. step**			
Neuroticism	-.32^c^	.10^c^	
**2. step**			
Autonomous motives	.08^a^	.12	.02^b^
Controlling motives	-.13^b^		
**3. step**			
Subjective well-being	.19^c^	.15	.03^b^
**4. step**			
Autonomous motives x Subjective well-being	.16^c^	.17	.02^c^
Controlling motives x Subjective well-being	.02		

^a^ p< .050

^b^ p< .010

^c^ p<.001

^d^ Beta-how many standard deviations a dependent variable will change, per standard deviation increase in the predictor variable

^e^ R^2^- proportion of variance in the criterion variable explained by the predictor variable

^f^ ΔR2- difference in R^2^ between previous and current step of the analysis

**Fig 1:**
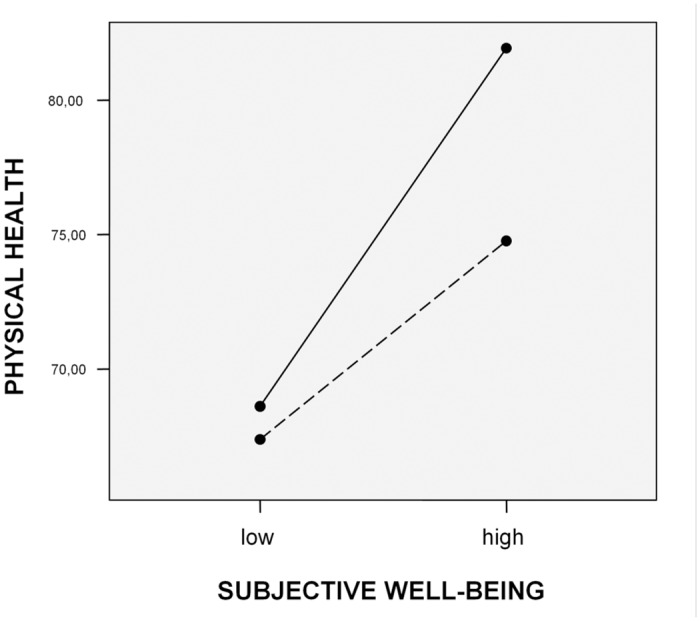
High RAI and high subjective well-being contribute to physical health. RAI …. low, **___** high.

To further clarify the effects of significant interactions between RAI (or autonomous motives) and subjective well-being on physical health, 2x2 analyses of variance were performed. Independent variables (RAI, autonomous motives and subjective well-being) were divided into groups of high and low scores. In order to do that 30% of the lowest and 30% of the highest values were set aside from the distribution of results. Illustration of the moderation effect of RAI on the relationship between subjective well-being and physical health can be found in Fig 1. As it can be seen the best physical health was reported by subjects with high subjective well-being and high RAI. In contrast, poorer health was found among those with high well-being and low RAI. Thus, the higher the degree of self-determination, the greater the positive impact of high well-being on physical health.

**Fig 2:**
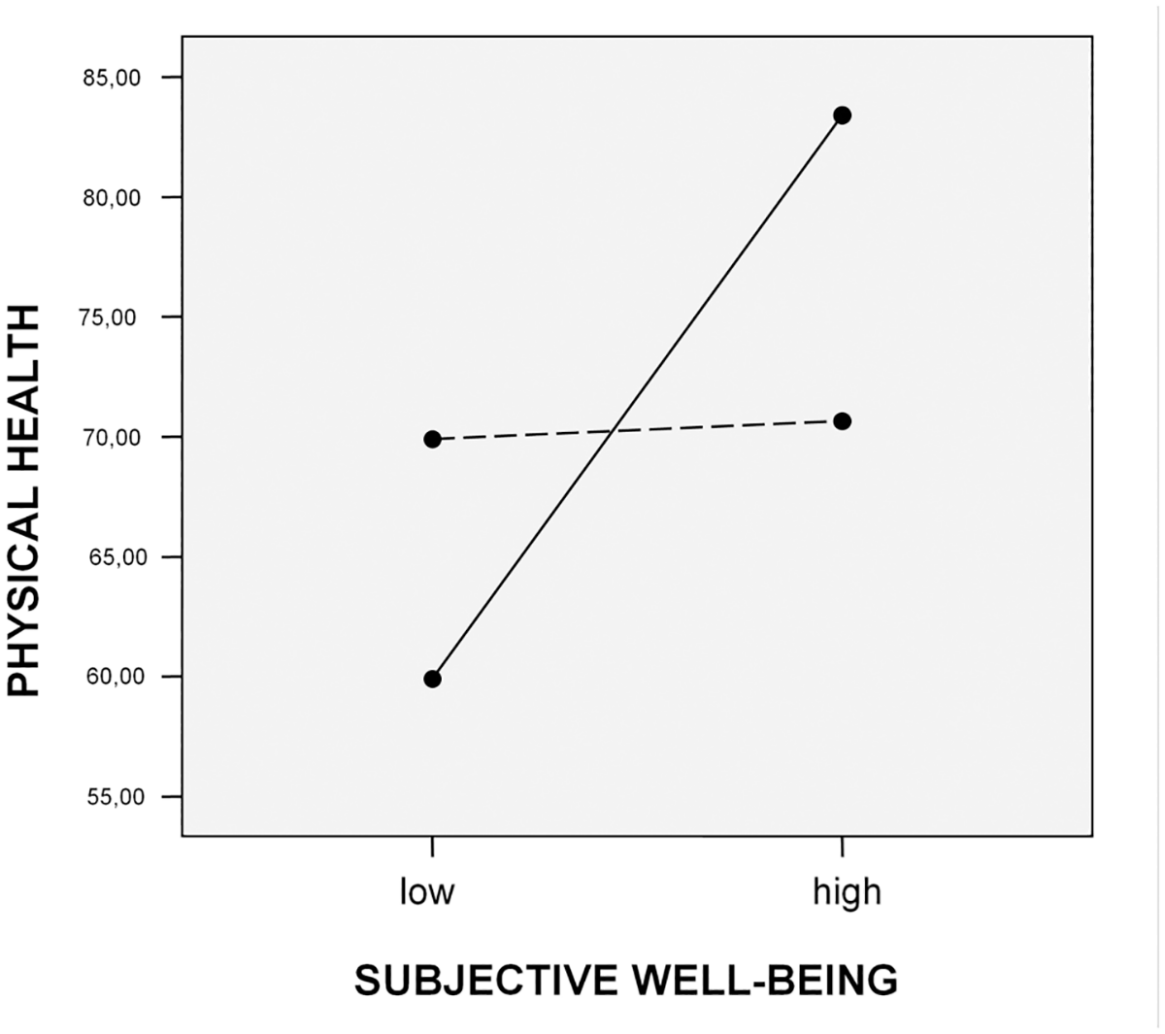
High autonomous motives and high subjective well-being contribute to physical health. Autonomous motives …. low, ___ high.

Moderator effect of autonomous motives on the association between subjective well-being and physical health is shown in Fig 2. The best physical health was found among subjects whose behaviour was initiated by high level of autonomous motives and a high level of subjective well-being. In comparison, poorer health evidenced subjects with a low level of autonomous motives and high level of subjective well-being. Interestingly, the least healthy were those with a high level of autonomous motives but a low level of subjective well-being.

Next, the moderating role of RAI (or autonomous and controlling motives) on the relationship between components of subjective well-being and physical health was tested. Here again, a hierarchical regression analysis was performed. The predictor variables were neuroticism, RAI and components of subjective well-being (positive affect, negative affect and satisfaction with goals). In the first step of analysis neuroticism was entered, in the second RAI, in the third the components of subjective well-being, and in the fourth the interaction between RAI and the components of subjective well-being. In additional analyses, instead of RAI autonomous and controlling motives were introduced. Tables 4 and 5 presents results of these analyses. The results have shown that the interaction between RAI and components of well-being did not impact physical health, concluding that self-determination is not a moderator of the relationship between components of well-being and physical health. On the other hand, the effect of the interaction between autonomous motives and positive affect on physical health was significant, which suggests the moderating role of autonomous motives in the association between positive affect and somatic health.

**Table 4:** The results of regression analyses in which RAI was tested as the moderator of the relationship between components of subjective well-being and physical health.

PHYSICAL HEALTH			
Predictors	Beta^b^	R^2^ ^c^	ΔR2^d^
**1. step**			
Neuroticism	-.32^a^	.10^a^	
**2. step**			
RAI	.17^a^	.13	.03^a^
**3. step**			
PA	.02	.16	.03^a^
NA	-.20^a^		
Satisfaction with goals	.03		
**4. step**			
RAI x PA	.07	.17	.01
RAI x NA	-.12		
RAI x Satisfaction with goals	.04		

^a^ p<.001

^b^ Beta-how many standard deviations a dependent variable will change, per standard deviation increase in the predictor variable

^c^ R^2^- proportion of variance in the criterion variable explained by the predictor variable

^d^ ΔR2- difference in R^2^ between previous and current step of the analysis

**Table 5:** The results of regression analyses in which autonomous and controlling motives were tested as the moderators of the relationship between components of subjective well-being and physical health.

PHYSICAL HEALTH			
Predictors	Beta^d^	R^2^ ^e^	ΔR2^f^
**1. step**			
Neuroticism	-.32^c^	.10^c^	
**2. step**			
Autonomous motives	.08^a^	.12	.02^b^
Controlling motives	-.13^b^		
**3. step**			
PA	.02	.16	.04^c^
NA	-.22^c^		
Satisfaction with goals	.03		
**4. step**			
Autonomous motives x PA	.09^a^	.19	.03^b^
Autonomous motives x NA	-.51		
Autonomous motives x Satisfaction with goals	.05		
Controlling goals x PA	-.04		
Controlling goals x NA	-.32		
Controlling goals x Satisfaction with goals	-.04		

^a^ p< .050

^b^ p< .010

^c^ p<.001

^d^ Beta-how many standard deviations a dependent variable will change, per standard deviation increase in the predictor variable

^e^ R^2^- proportion of variance in the criterion variable explained by the predictor variable

^f^ ΔR2- difference in R^2^ between previous and current step of the analysis

**Fig 3:**
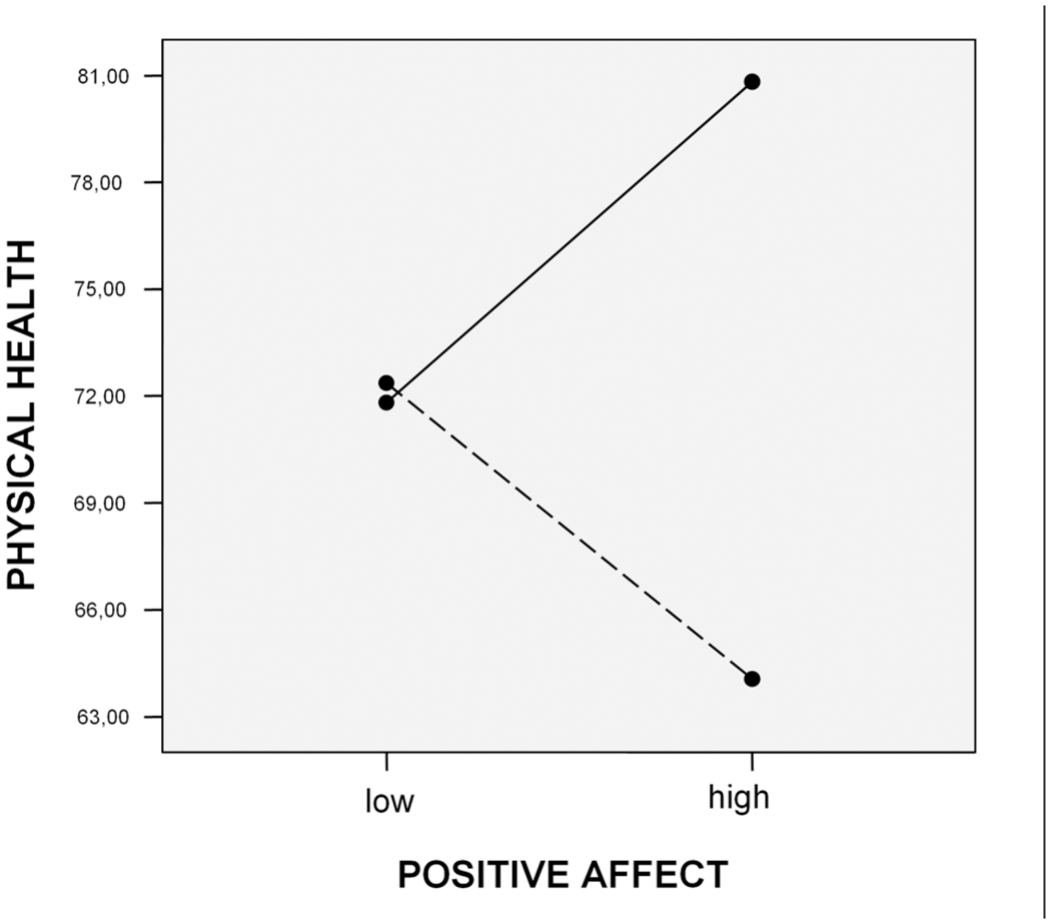
High autonomous motives and high positive affect contribute to physical health. Autonomous motives …. low, ___ high.

To further our understanding of how interaction between autonomous motives and positive affect impacts health, 2x2 analysis of variance was performed. Once again, independent variables were divided according to the criteria of 30% of the highest/lowest results in the distribution. Fig 3 displays a graphic illustration of the moderator effect of autonomous motives on the association between positive affect and physical health. As it can be seen, the best physical health was reported by participants who had high level of positive affect and behaviors initiated by a high degree of autonomous reasons. On the other side, the worst physical health had subjects with high positive affect and behaviours regulated by low level of autonomous motives.

## Discussion

The main hypothesis of this study was that autonomy would amplify positive effects of high well-being on physical health. Results provide support for this proposition. It has been shown that experiential autonomy moderates the relationship between subjective well-being and physical health. The results suggest that higher the degree of self-determination, greater is the positive contribution of high well-being to physical health. Accordingly, subjects with high self-determination and high level of well-being had good physical health while in comparison to them, health of people with a high level of well-being but a low self-determination was poorer.

The results of additional analysis demonstrated that the moderating role of the self-determination is based on the moderational effect of autonomous motives and not the controlling ones. Consequently, a good physical health had subjects with a high well-being and behaviours initiated by high level of autonomous motives. In relation to them, the health of the participants with high well-being but a low level of autonomous motives was somewhat poorer. Particularly interesting was to find that the worst physical health was reported by students with low subjective well-being and behaviours regulated by a high degree of autonomous motives.

Component-level analysis has shown that motives moderate the relationship between physical health and one component of subjective well-being, positive affect. The extent to which positive effect contributed to physical health was determined by the level of autonomous motives. Accordingly, best somatic health was registered among students with high positive affect and behaviours regulated by high degree of autonomous motives. On the contrary, very poor health was found among subjects with high levels of positive affect, but behaviours regulated by a low degree of autonomous motives.

Looking at those results it is important to highlight the extent to which moderational effects of motives found in this study are important. Generally speaking, the interaction effects between the moderator and predictor variables are, in relation to direct effects of predictors on the criterion variable, relatively small or non-significant. So the fact that the interactions between motives and well-being have significant contribution to health above and beyond neuroticism, a personality trait that is accountable for the strong relationship between subjective well-being and self-rated health, is an indication of the strength of their effects or their robustness. Therefore, the finding that the interactions between motives and well-being contributes to physical health after control of neuroticism, speaks a lot.

The results of this study are in contradiction to the traditional approach which holds that well-being, the subjective measures of physical health, and their relationship are determined by personality [28]. In contrast, this research suggests that the motives and interests of an individual for which activity is initiated, offer a better explanation when will well-being positively or negatively impact physical health. Overall, present findings suggest that optimal functioning of the individual is based on a complete dedication to the life goals that one sets for oneself, rather than the mere attainment of desires that one feels are alien to his true self. This indicates that any behaviour that is directed towards one's development and progress has a significant positive effect on health. The results also highlight that where there is a desire to achieve something deeply valuable to a person then a low well-being, which comes as a consequence of not being successful in such pursuits, is particularly detrimental to somatic health. This is an indication of the extent to which it is in human nature to seek as well as achieve meaning and purpose in one's life but if not successful in this we can be, so to speak, self—destructive when it comes to our own health. That infers that finding a meaning in life is central to health. Although this premise is rarely present in the psychological literature, there are some models and data which support it. For example, a key concept in the Antonovsky's salutogenic model [45] is a sense of coherence. Sense of coherence is constituted of three core components called comprehensibility, manageability and meaningfulness. In this model, meaningfulness refers to the extent to which one feels that life makes sense emotionally. According to Antonovsky sense of coherence contributes to flexible problem solving and coping in the time of stress. The stronger the sense of coherence, the greater is the mobilization of appropriate resources during times of hardship which in turn, positively affects health [45]. Now, one of the best examples of the extent to which meaningfulness contributes to health is the information related to survivors of Nazi concentration camps. What differentiated those who survived compared to those who did not, was the presence of meaning in life [46]. Thus, meaning in life plays an important role in the adjustment and health of the individual, not only in everyday situations, but also in situations that are extremely threatening.

The answers to the question as to why positive mental states tied to the autonomous behaviours have such a large role in physical health really need to be sought in the differences between a hedonic compared to a eudaimonic approach to life and in the goals one is trying to achieve with each lifestyle.

According to a hedonic approach, the goal that one strives to achieve is a high level of pleasure, the higher the better. The source of pleasure is not important and the individual tries in various ways and at any cost to achieve it.

In contrast, the goal of a eudaimonic approach is the development of oneself, use of one's own potential in order to contribute to the greater meaning and purpose of life. The aim of this approach is the investment of effort, the use of abilities and skills in order to achieve one's "real self". So the positive psychological state, one that arises out of those pursuits, occurs spontaneously and is not a goal in itself [47]. The assumption is that only such energetic positive state, the one that is an outcome of such a proactive way of life, can contribute to greater somatic health. One reason for this is that a strong sense of authenticity, initiated by high experiential autonomy, contributes to greater integration of cognitive and behavioral processes into one comprehensive self whereby, on the level of manifestation, this greater personality integration is expressed in the form of self-actualization and better health. Some studies provide support for this. Sheldon, Ryan, Rawsthorne and Ilardi [48] have found that different constructs that indicate greater personality integration, such as high authenticity, a low degree of conflict between roles and a higher degree of integration between different parts of self, contribute to well-being as well as to mental and physical health.

Another reason for the positive effects of autonomy on physical health may be in the high level of energy that accompanies autonomous actions. Autonomous behaviors are associated with setting clear goals, the power of intention and a sense of challenge and competence for the purpose of challenging one's own possibilities and potential. As results, such endeavours are accompanied by a high level of energy [49] which is interpreted as a sign of good health. After all, SDT suggests that vitality or the energy available to the self is one of the best indicators of optimal functioning of the individual. It is argued that vitality depends upon the motivational aspects of behaviour specifically, on the extent to what behavior is autonomous. Nix, Ryan, Manly and Deci [50] have shown that activities regulated by controlling motives contribute to positive affect but not vitality, while autonomously regulated activities increase both, positive affect and vitality. Research also shows that vitality is closely linked to positive psychological and physical states while ego depletion is associated with negative emotions and ill health [51]

Aside from this, it is possible that high autonomy advances physical health through more adaptive coping strategies employed during time of stress. Fredrickson [52] argued that positive emotions broaden though-action repertoire during exposure to stressors. It is well-documented that positive psychological states contribute to flexible, creative and functional ways of thinking which enables one to see the world from the wider perspective [53]. This suggests that self-determined individuals handle life stressors in more adaptable ways which creates better living conditions for them.

The positive impact of the autonomy on the physical health can also be explained via immune responses. It is commonly known that negative states and chronic stress activate the fight-flight response. During this reaction all the physiological systems are fully activated so an organism can deal and manage stressor successfully. However, chronic exposure to stress, which is present if an individual is living life non-autonomously, can lead to wear-and-tear of the body. In other words, it can lead to detrimental health. On the other side, positive mental states stimulate positive regulation of immune functions [54] and mental states associated with high autonomy have more pronounced effects on immune system than psychological states linked to activities that are low in autonomous motivation [12].

All the findings should be observed in the light of limitations of this study. First of all, as research was carried out on a sample of students, a question arises whether results can be generalized to people of other age, levels of education or lifestyles. Secondly, as we are dealing with correlation and cross-sectional research, drawing conclusions in terms of the cause and effect is not possible so longitudinal research is needed in order to comment results in those terms with greater certainty. Thirdly, self-reports were used as method of assessment of well-being and somatic health which is not the best choice since self-reports are known to be contaminated by bias. So, future research with alternative means of testing these constructs is needed.

Unlike any other previous research, this study has looked into the complex interplay between motivation, well-being and physical aspects of human functioning to answer whether the magnitude of the effect that well-being has on physical health depends on the experiential autonomy. It was demonstrated that the only way to gain understanding of the conditions under which subjective well-being positively or negatively affect somatic health is by investigating the motivational features of behaviour. By identifying this moderator we are in position to gain further understanding of human health in its broader sense. It all points to conclusion that people are made in such a way that they can fulfill their own potential and achieve state of health, and that all activities and psychological states which are consistent with such tendencies contribute to the development of an organism in that direction. Recommendation for future research involves exploration of how the interaction between autonomy and well-being impacts mortality, immune functions or survival rates for people who are living with serious health conditions. Research alike is needed in order to gain further knowledge on how life choices affect physical health. In that way we can learn more about health promotion and disease prevention strategies.
